# Effect of low-dose aspirin on urinary 11-dehydro-thromboxane B2 in the ASCEND (A Study of Cardiovascular Events iN Diabetes) randomized controlled trial

**DOI:** 10.1186/s13063-023-07198-z

**Published:** 2023-03-04

**Authors:** Sarah Parish, Georgina Buck, Theingi Aung, Marion Mafham, Sarah Clark, Michael R. Hill, Rory Collins, Louise Bowman, Jane Armitage, on behalf of the ASCEND Study Collaborative Group

**Affiliations:** 1 MRC Population Health Research Unit, Nuffield Department of Population Health, University of Oxford, Big Data Institute, Old Road Campus, Roosevelt Drive, Oxford, OX3 7LF UK; 2 Clinical Trial Service Unit and Epidemiological Studies Unit, Nuffield Department of Population Health, University of Oxford, Oxford, UK

**Keywords:** Randomized placebo-controlled trial, Daily low-dose aspirin, 11-Dehydro-thromboxane B2, Diabetes

## Abstract

**Background:**

Aspirin is widely used for cardioprotection with its antiplatelet effects due to the blocking of thromboxane A2 production. However, it has been suggested that platelet abnormalities in those with diabetes prevent adequate suppression with once daily aspirin.

**Methods:**

In the ASCEND randomized double-blind trial of aspirin 100 mg once daily versus placebo in participants with diabetes but no history of cardiovascular disease, suppression was assessed by measuring 11-dehydro-thromboxane B2 excretion in urine (U-TXM) in a randomly selected sample of 152 participants (76 aspirin arm, 74 placebo arm), plus 198 (93 aspirin arm, 105 placebo arm) adherent to study drugs and selected to maximize the numbers ingesting their last tablet 12–24 h before urine sampling. U-TXM was assayed using a competitive ELISA assay in samples mailed a mean of 2 years after randomization, with time since taking last aspirin/placebo tablet recorded at the time of sample provision. Effective suppression (U-TXM < 1500 pg/mg creatinine) and percentage reductions in U-TXM by aspirin allocation were compared.

**Results:**

In the random sample, U-TXM was 71% (95% CI 64–76%) lower among aspirin vs placebo-allocated participants. Among adherent participants in the aspirin arm, U-TXM was 72% (95% CI 69–75%) lower than in the placebo arm and 77% achieved effective suppression overall. Suppression was similar among those who ingested their last tablet more than 12 h before urine sampling with levels in the aspirin arm 72% (95% CI 67–77%) lower than in the placebo arm and 70% achieving effective suppression.

**Conclusions:**

Daily aspirin significantly reduces U-TXM in participants with diabetes, including at 12–24 h after ingestion.

**Trial registration:**

ISRCTN ISRCTN60635500. Registered on 1 Sept 2005; ClinicalTrials.gov NCT00135226. Registered on 24 Aug 2005.

**Supplementary Information:**

The online version contains supplementary material available at 10.1186/s13063-023-07198-z.

## Introduction

Aspirin is widely used for cardioprotection, with its antiplatelet effects due to the irreversible blocking of thromboxane A2 production in platelets for their ~ 10-day lifetime [1]. Thromboxane A2 inhibition can be assessed by measuring the urinary excretion of 11-dehydro-thromboxane B2 (U-TXM), a stable metabolite of thromboxane A2 [2, 3]. However, in people with diabetes, platelet abnormalities may mean that inhibition is shorter lasting, leading to the suggestion that twice daily aspirin may be needed in these patients [4, 5].

Therefore, we assessed the effectiveness of once daily aspirin to suppress U-TXM, particularly at 12–24 h after ingestion, in the context of the ASCEND (A Study of Cardiovascular Events iN Diabetes) randomized trial of daily low-dose aspirin in people with diabetes [6, 7].

## Methods

The ASCEND 2 × 2 factorial randomized trial, in 15,480 people with diabetes but no occlusive arterial disease, investigated the effects of 6–10 years of aspirin 100 mg once daily versus placebo tablets and omega-3 fatty acids 1 g once daily versus placebo capsules on cardiovascular events, cancer, and bleeding (protocol and CONSORT diagram [7]; Data Analysis Plan [6]). During the 2-month placebo “run-in” phase of the trial, baseline blood and spot urine samples were collected locally in general practice surgeries and mailed to the central laboratory. Further blood and urine samples were collected by mail at a mean of 2 years after randomization in a random sample of around 10% of participants. The time a participant last took their (aspirin/placebo) tablet and gave their sample was recorded.

A random subgroup of 152 participants (balanced by treatment allocation) with urine samples at both baseline and follow-up was selected for the U-TXM assay. This was estimated conservatively to give 95% confidence limits (CIs) of about ± 10% or less around an anticipated 60–70% reduction in U-TXM in aspirin versus placebo-allocated participants (Supplementary Methods). U-TXM at follow-up was later assayed in a further 198 participants who reported being adherent to their study tablets (the 98 who reported taking their tablet more than 12 h before their sample and 100 selected at random).

### Laboratory assays

U-TXM in previously frozen aliquots of the urine samples was assayed in duplicate using a competitive ELISA (AspirinWorks® test kit, Corgenix, Peterborough, UK) as used in several previous studies [5, 8]. U-TXM is divided by creatinine concentration from spot urine samples to make 24-h collection of urine unnecessary, giving a U-TXM value reported as pg/mg creatinine, with U-TXM < 1500 pg/mg creatinine by this assay taken as indicating effective suppression [9] (see Supplementary Methods for further details of the assay procedures).

Assay values below or above the linear range after possible dilutions were imputed with the respective assay limit. The Pearson correlation between the U-TXM duplicate values where both were present was very high (0.98). The 2 participants with a baseline level in both duplicate measurements above the assay range after twofold dilution were excluded as estimation of any reduction would not be accurate. Imputed follow-up values (13 participants had both duplicate measurements imputed) were not excluded as this would cause bias (with high values more likely on placebo and low values on aspirin). The average of the U-TXM values across the duplicates (after imputation) divided by the creatinine was used as the sample result.

### Statistical analysis

The relationship between follow-up and baseline U-TXM was plotted (on a log scale), distinguishing categories by adherence to study aspirin/placebo tablets (reported on the sampling form) and non-study aspirin use. Participants were classified as non-adherent (last tablet date earlier than the day before sampling, or known to be taking non-study aspirin); last tablet taken ≤ 12 h before sample; last tablet taken > 12 h before sample; and adherent but the time of taking tablet not known (distribution of timings shown in Fig. S1). Two participants could not be classified.

Comparisons by intention-to-treat, and restricting by adherence, were of mean log U-TXM at follow-up by aspirin allocation (without adjustment for baseline values). A preliminary analysis had shown that the Pearson correlation between log U-TXM measurements at baseline and follow-up among adherent participants in the placebo arm was weak (0.48), indicating little improvement would be gained by adjustment for baseline levels (and hence baseline samples were not assayed for the additional adherent sample participants) [10]. Effective suppression and differences in log U-TXM by treatment allocation were analyzed by logistic and linear regression respectively. Differences, *d*, in log U-TXM were expressed as percentage reductions in U-TXM using 100 (1-exp(*d*)).

## Results

Baseline characteristics were found to be well balanced between the two randomized arms in the overall population (Tables 1, S1, and S2 of the main aspirin paper [7]) and reasonably well balanced in the U-TXM samples (Supplementary Table S1).

**Fig. 1:**
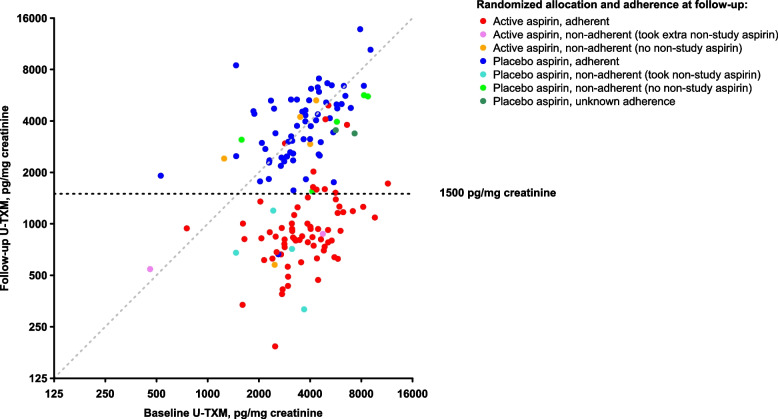
Urinary 11-dehydro-thromboxane B2 (U-TXM) during follow-up versus at baseline by aspirin allocation and use in the random sample

During follow-up, in the intention-to-treat analysis of the random sample, 82% allocated aspirin versus 7% allocated placebo achieved effective suppression of U-TXM (Table 1). Among participants reporting adherence to aspirin, 86% in the random sample and 71% in the additional adherent sample achieved suppression, 3 participants in the random sample had no apparent suppression (Fig. 1), and 4 participants in the additional adherent sample had high values, while most of the other 30 above the effective suppression level had follow-up levels below 3000 pg/mg, suggesting partial suppression (Fig. 1, Fig. S2). Only 3% of participants adherent to placebo had effective suppression of U-TXM at follow-up.

**Table 1:** Suppression of urinary 11-dehydro-thromboxane B2 (U-TXM) at follow-up with allocation to daily low-dose aspirin

		Percentage achieving effective suppression* by treatment allocation		Geometric mean U-TXM (95% CI) by treatment allocation		Reduction in U-TXM with aspirin (95% CI)
Group	*N*	Aspirin	Placebo	Aspirin	Placebo	
Random sample						
All	150	62/76 (82%)	5/74 (7%)^†^	979 (854 − 1122)	3322 (2874 − 3839)	71% (64 to 76%)^†^
Non-adherent	16	3/7 (43%)	4/9 (44%)^‡^	1712 (845 − 3468)	1686 (860 − 3305)	− 2% (− 82 to 43%)^‡^
Adherent	132	59/69 (86%)	1/63 (2%)	925 (814 − 1050)	3655 (3221 − 4147)	75% (69 to 79%)
Time of ingestion relative to sample						
≤ 12 h before	100	45/52 (87%)	1/48 (2%)	896 (768 − 1044)	3517 (3039 − 4070)	75% (69 to 79%)
> 12 h before	28	12/14 (86%)	0/14 (0%)	1015 (801 − 1285)	4104 (3139 − 5365)	75% (63 to 83%)
Unknown	4	2/3 (67%)	0/1 (0%)	1042 (536 − 2024)	4563	77% (14 to 94%)
Adherent sample						
Adherent	198	66/93 (71%)	4/105 (4%)	1366 (1233 − 1513)	4511 (4114 − 4947)	70% (65 to 74%)
Time of ingestion relative to sample						
≤ 12 h before	100	38/50 (76%)	2/50 (4%)	1390 (1220 − 1584)	4286 (3781 − 4860)	68% (61 to 73%)
> 12 h before	98	28/43 (65%)	2/55 (4%)	1338 (1137 − 1576)	4726 (4134 − 5403)	72% (66 to 77%)
Either sample						
Adherent	330	125/162 (77%)	5/168 (3%)	1157 (1062 − 1260)	4169 (3864 − 4498)	72% (69 to 75%)
Time of ingestion relative to sample						
≤ 12 h before	200	83/102 (81%)	3/98 (3%)^§^	1111 (996 − 1239)	3891 (3529 − 4289)	71% (67 to 75%)^§^
> 12 h before	126	40/57 (70%)	2/69 (3%)	1250 (1088 − 1436)	4593 (4075 − 5176)	72% (67 to 77%)
Unknown	4	2/3 (67%)	0/1 (0%)	1042 (536 − 2024)	4563	77% (14 to 94%)

*CI* confidence interval, *U-TXM* urinary 11-dehydro-thromboxane B2 (pg/mg creatinine). *Effective suppression = U-TXM < 1500 pg/mg creatinine

^†^*P* < 0.0001 for difference by aspirin vs placebo

^‡^*P* < 0.0001 for heterogeneity in the difference by adherence to randomized treatment

^§^*P* > 0.5 for heterogeneity in the difference by ≤ 12 versus > 12 h from ingestion to urine sample in those adherent to randomized treatment

In adherent participants, there were no statistically significant differences between those who ingested their last tablet ≤ 12 (mean 3.0 [SE 0.2]) hours versus > 12 (mean 18.2 [SE 0.3]) hours before urine sampling in the percentages achieving effective suppression with aspirin (81% versus 70%, 77% overall) or in the lowering of U-TXM with aspirin (71%, [95% CI 67–75%] versus 72% [95% 67–77%], 72% [95% 69–75%] overall). Adjustment for participant age did not alter estimates of the reduction in U-TXM with aspirin or the statistical significance of the findings (data not shown).

## Discussion

Among people with diabetes, adherence to daily low-dose aspirin reduced U-TXM by 72%. This reduction was similar to reductions reported in a previous longitudinal daily aspirin intervention comparison in people with and without diabetes [8] and in a randomized crossover trial among people with diabetes [5]. Suppression was achieved in 77% of participants in the present study, similar to the 85% reported in those with diabetes in the study by Ames et al. [8]. However, in that study, U-TXM levels were about 50% higher in participants with diabetes than in non-diabetic controls and so, with a similar percentage reduction in both groups, the percentage effectively suppressed was higher (92%) in the healthy control group after aspirin. It has been suggested that > 95% U-TXM inhibition may be needed to achieve full platelet inhibition [1]. Nevertheless, the finding of a statistically significant 12% proportional reduction in the primary cardiovascular outcome with aspirin in ASCEND shows that the suppression achieved was sufficient to be beneficial [7].

The present study also investigated the level of suppression by time since taking aspirin. Among adherent participants taking their tablet > 12 h before their urine sample, the reduction in U-TXM (72%) and percentage achieving effective suppression (70%) were similar to those in the study overall. However, a limitation of the present study was that it did not include different dosing schedules. A randomized crossover trial in 24 diabetic participants found a somewhat greater reduction in mean U-TXM with 100 mg aspirin twice daily (80% reduction) than once daily (76% reduction; difference between regimens statistically significant at *P* = 0.05), while with 200 mg once daily the reduction was intermediate (77%, but not statistically significantly different from either of the other regimes) [5]. Serum thromboxane recovery after aspirin dosing has also been found to vary between people but be resolved by twice daily dosing [11].

## Conclusions

Among people with diabetes taking daily low-dose aspirin versus placebo, the reduction in U-TXM was similar to that seen in other diabetic and non-diabetic populations. There was no evidence of substantive deterioration in suppression over 24 h and the aspirin regimen in ASCEND resulted in statistically significant cardioprotection [7]. Nevertheless, only 77% of participants adherent to aspirin achieved effective suppression, and therefore, it remains possible that a higher total dose of aspirin, given either once or twice daily, might achieve even more effective suppression in some people with diabetes.

## Supplementary Information

**Additional file1:** **Table S1.** Baseline characteristics by randomized treatment allocation. **Figure S1.** Hours between ingestion of last tablet and urine sample in adherent participants. **Figure S2.** Urinary 11-dehydro thromboxane B2 (U-TXM) during follow-up by aspirin allocation in the adherent sample. Points have been randomly spread in the x-direction for clarity.

**Additional file 2.** Analysis dataset containing all the information needed to replicate this analysis, with one row per person.

## Data Availability

All data analyzed during this current study are included in the Supplementary information files of this article.

## Abbreviation

U-TXM: Urinary excretion of 11-dehydro-thromboxane B2
