# Supplementary material for: Effect of low-dose aspirin on urinary 11-dehydro-thromboxane B2 in the ASCEND (A Study of Cardiovascular Events iN Diabetes) randomized controlled trial
Source: Trials. 2023 Mar 4;24:166. doi: 10.1186/s13063-023-07198-z (PMC9985834; doi:10.1186/s13063-023-07198-z)
Supplement: Supplementary file 1 — Additional file1: Table S1. Baseline characteristics by randomized treatment allocation. Figure S1. Hours between ingestion of last tablet and urine sample in adherent participants. Figure S2. Urinary 11-dehydro thromboxane B2 (U-TXM) during follow-up by aspirin allocation in the adherent sample. Points have been randomly spread in the x-direction for clarity. [file 13063_2023_7198_MOESM1_ESM.pdf]

Supplementary Materials

Table of contents

Supplementary Methods		2
Supplementary References		4
Supplementary Table		
Table S1	Baseline characteristics by randomized treatment allocation	5
Supplementary Figures		
Figure S1	Hours between ingestion of last tablet and urine sample in adherent participants	6
Figure S2	Urinary 11-dehydro thromboxane B2 (U-TXM) during follow-up by aspirin allocation in the adherent sample	7

Supplementary Methods

Size of the random sample

Based on limited information available from prior studies when the study was planned in 2014[1,2] we assumed that urinary 11-dehydro-thromboxane B2 (U-TXM) was log-normally distributed and that the standard deviation of both log U-TXM and of the difference in log U-TXM between baseline and follow-up were 0.7- 0.8. We anticipated, with allowance for uncertainty and non-adherence, that randomisation to aspirin might reduce U-TXM by 60-70%. Conservatively assuming a standard deviation of 0.8 and a 60% reduction, 75 participants per aspirin/placebo arm were needed to provide an expected confidence interval of about +/- 10% around the estimated reduction.

Additional details of assay procedures

Urine creatinine was assayed using a Beckman-Coulter DxC800 clinical chemistry analyser and manufacturers' reagents, calibrators and settings (Beckman-Coulter, UK). The Wolfson laboratory is accredited to ISO 10725:2017 as a testing laboratory (UK Accreditation Service No. 2799), and urine creatinine is on their schedule of accreditation.

Samples selected for U-TXM assay were restricted to those that had been received by the laboratory 1-2 days after the sample was taken and excluded those with urine creatinine >17.7mmol/l, as previous validation work by the laboratory indicated an increase in false U-TXM results from samples with particularly concentrated urine. U-TXM was assayed using a Corgenix AspirinWorks® ELISA test following manufacturers' instructions. This assay was developed jointly by three companies

(Corgenix, Denver, USA, Creative Clinical Concepts, Denver USA and Cayman Chemical, Ann Arbour, USA) and was chosen because it has been used in a number of other studies.[1-3]

The standard process for the assay was a factor of 5 dilution for samples.[4,5] To measure values outside the linear working range of 300 pg/ml to 4000 pg/ml alternative dilutions were performed where necessary and there was sufficient sample. For the random sample, dilutions of 0.5 and 2 were additionally considered; for the adherent sample, dilutions of 0.4 and 2 were additionally considered. A correction factor for the Reference Solution of 1.07 in the random sample and 1.02 in the adherent sample was applied as specified in the kit instructions at the time of the two assay batches.

Participants with both baseline measurements above the upper linear working range of the assay after dilution were excluded. Other measurements below or above the linear working range of the assay, were imputed as the respective assay limit (following which the adjustments for the dilution and correction factor were applied). The average of the U-TXM values across the duplicates (after imputation) divided by the creatinine was used as the sample result in pg/mg creatinine.

Supplementary References

1. Ames PR, Batuca JR, Muncy IJ, et al. Aspirin insensitive thromboxane generation is associated with oxidative stress in type 2 diabetes mellitus. *Thromb Res*. 2012;130:350-354. doi: 10.1016/j.thromres.2012.03.025
2. Eikelboom JW, Hirsh J, Weitz JI, et al. Aspirin-resistant thromboxane biosynthesis and the risk of myocardial infarction, stroke, or cardiovascular death in patients at high risk for cardiovascular events. *Circulation*. 2002;105:1650-1655. doi: 10.1161/01.cir.0000013777.21160.07
3. Bethel MA, Harrison P, Sourij H, et al. Randomized controlled trial comparing impact on platelet reactivity of twice-daily with once-daily aspirin in people with Type 2 diabetes. *Diabet Med*. 2016;33:224-230. doi: 10.1111/dme.12828
4. Administration UFaD. 510(k) SUBSTANTIAL EQUIVALENCE DETERMINATION DECISION SUMMARY ASSAY ONLY TEMPLATE: AspirinWorks Test Kit https://www.accessdata.fda.gov/cdrh_docs/reviews/K062025.pdf. Accessed 6 June 2022.
5. IBL International CC. Aspirin-Effect Detection Kit. https://novamedline.com/files/d039d453-778d-465e-a9fc-2794d5d62ebc.pdf. Accessed 6 June 2022.

Table S1: Baseline characteristics by randomized treatment allocation

	Randomized treatment allocation	
**Baseline characteristic**	**Aspirin arm**	**Placebo arm**
Random sample - N	76	74
Age at baseline in years - N (%)		
<60	19 (25.0%)	16 (21.6%)
60 to <70	31 (40.8%)	36 (48.6%)
≥70	26 (34.2%)	22 (29.7%)
Mean (SD)	65.4 (8.8)	65.3 (8.1)
Men - N (%)	52 (68.4%)	49 (66.2%)
Duration of diabetes in years - N (%)		
<9	35 (46.1%)	36 (48.6%)
≥9	38 (50.0%)	36 (48.6%)
Unknown	3 (3.9%)	2 (2.7%)
Median (IQR)	9 (6 - 16)	9 (4 - 13)
Adherent sample - N	93	105
Age at baseline in years - N (%)		
<60	27 (29.0%)	25 (23.8%)
60 to <70	49 (52.7%)	52 (49.5%)
≥70	17 (18.3%)	28 (26.7%)
Mean (SD)	63.2 (8.1)	65.5 (8.3)
Men - N (%)	49 (52.7%)	62 (59.0%)
Duration of diabetes in years - N (%)		
<9	56 (60.2%)	57 (54.3%)
≥9	37 (39.8%)	43 (41.0%)
Unknown	0 (0.0%)	5 (4.8%)
Median (IQR)	7 (4 - 12)	7 (3 - 15)
Adherent in either sample - N	162	168
Age at baseline in years - N (%)		
<60	45 (27.8%)	40 (23.8%)
60 to <70	77 (47.5%)	81 (48.2%)
≥70	40 (24.7%)	47 (28.0%)
Mean (SD)	64.0 (8.5)	65.3 (8.3)
Men - N (%)	97 (59.9%)	103 (61.3%)
Duration of diabetes in years - N (%)		
<9	88 (54.3%)	88 (52.4%)
≥9	71 (43.8%)	73 (43.5%)
Unknown	3 (1.9%)	7 (4.2%)
Median (IQR)	8 (4 - 15)	8 (3 - 13)

N=number of participants, SD=Standard deviation, IQR=Inter-quartile range.

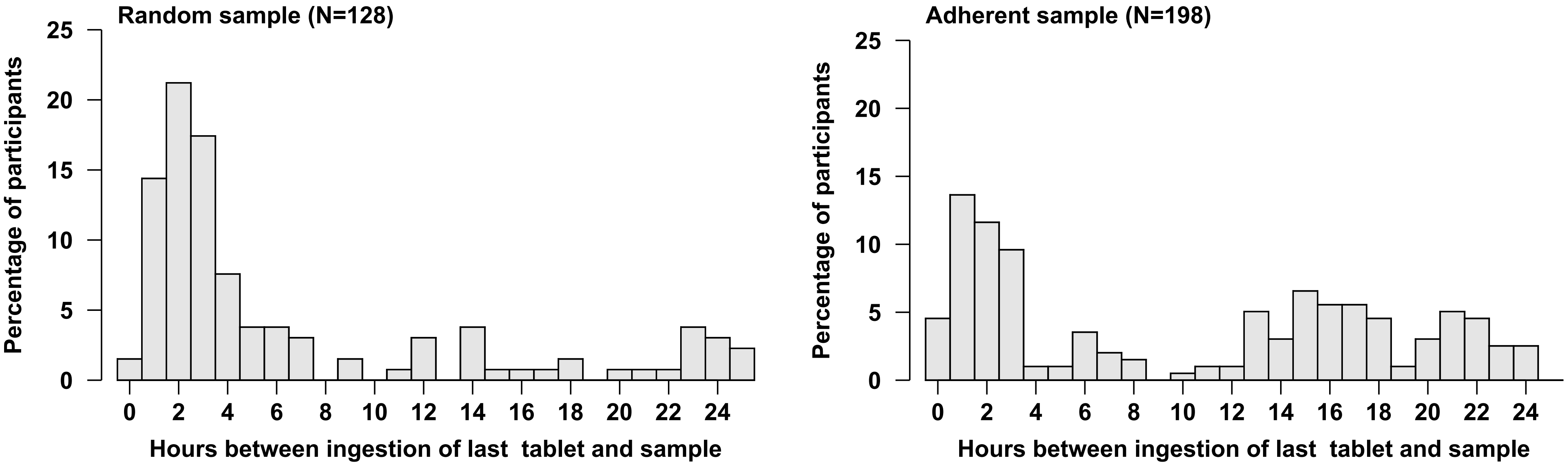

	Hours between ingestion of last tablet and urine sample					
	**≤ 12 hours**		**>12 hours**		**Overall**	
	**N**	**Mean (SE)**	**N**	**Mean (SE)**	**N**	**Mean (SE)**
**Random sample**	100	3.2 (0.2)	28	19.3 (0.9)	128	6.7 (0.6)
**Adherent sample**	100	2.9 (0.3)	98	17.8 (0.3)	198	10.3 (0.6)
**Either sample**	200	3.0 (0.2)	126	18.1 (0.3)	326	8.9 (0.4)

Figure S1: Hours between ingestion of last tablet and urine sample in adherent participants.

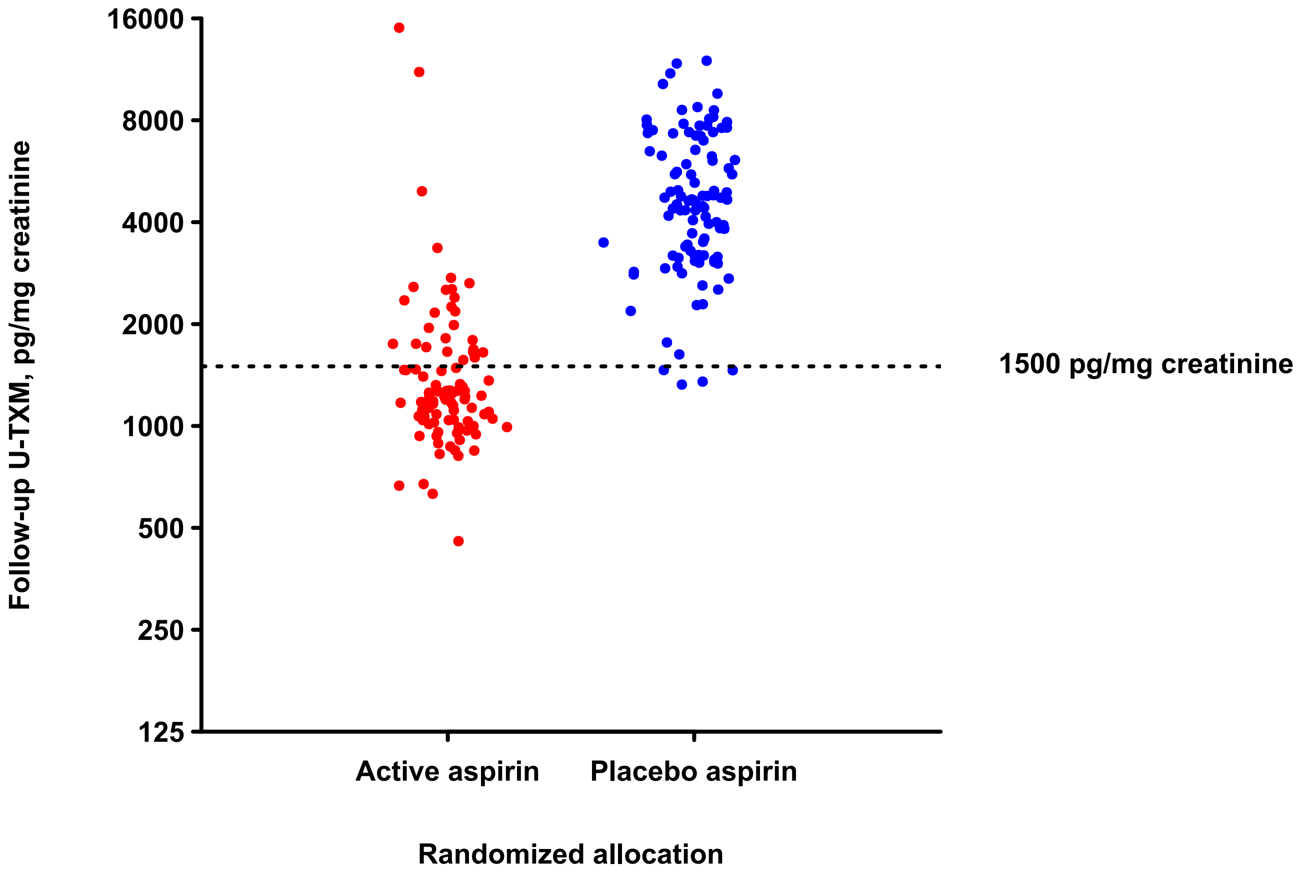

Figure S2: Urinary 11-dehydro thromboxane B2 (U-TXM) during follow-up by aspirin allocation in the adherent sample. Points have been randomly spread in the x-direction for clarity.
